# Impact of low pneumoperitoneum on renal function and acute kidney injury biomarkers during robot-assisted radical prostatectomy (RARP): a randomised clinical trial

**DOI:** 10.1007/s11701-023-01744-2

**Published:** 2024-01-17

**Authors:** Hayder Alhusseinawi, Lotte Sander, Aase Handberg, Rikke W. Rasmussen, Pernille S. Kingo, Jørgen B. Jensen, Sten Rasmussen

**Affiliations:** 1 Department of Clinical Medicine, Aalborg University, Aalborg, Denmark; 2 Department of Urology, Aalborg University Hospital, Aalborg, Denmark; 3 Department of Clinical Biochemistry, Aalborg University Hospital, Aalborg, Denmark; 4 Department of Clinical Medicine, Aarhus University, Aarhus, Denmark; 5 Department of Urology, Aarhus University Hospital, Aarhus, Denmark

**Keywords:** Low pneumoperitoneum, u-NGAL, Postoperative AKI, Renal injury Biomarkers

## Abstract

The objective of this study was to evaluate the effect of low pneumoperitoneum pressure (Pnp) on renal function and renal injury biomarkers during robot-assisted radical prostatectomy (RARP). A single-centre, triple-blinded, randomised clinical trial was conducted with 98 patients undergoing RARP, who were assigned to either standard Pnp of 12 mmHg or low Pnp of 7 mmHg. The primary outcome was urinary neutrophil gelatinase-associated lipocalin (u-NGAL), and several other kidney injury biomarkers were assessed as secondary outcomes. Acute kidney injury (AKI) was evaluated using the Kidney Disease Improving Global Outcomes (KDIGO) criteria, the gold standard method for defining AKI. The trial was registered on ClinicalTrials.gov (NCT04755452). Patients in the low Pnp group had significantly lower levels of u-NGAL (mean difference − 39.9, 95% CI − 73.7 to − 6.1, *p* = 0.02) compared to the standard Pnp group. No significant differences were observed for other urinary biomarkers. Interestingly, there was a significant difference in intraoperative urine production between the groups (low Pnp median: 200 mL, IQR: 100–325 vs. standard Pnp median: 100 mL, IQR: 50–200, *p* = 0.01). Similarly, total postoperative urine production also varied significantly (low Pnp median: 1325 mL, IQR: 1025–1800 vs. standard Pnp median: 1000 mL, IQR: 850–1287, *p* = 0.001). The occurrence of AKI, as defined by the KDIGO criteria, did not differ significantly between the groups. Low Pnp during RARP resulted in lower u-NGAL levels, suggesting a potential benefit in terms of reduced renal injury. However, the lack of a notable difference in AKI as defined by the KDIGO criteria indicates that the clinical significance of this finding may be limited. Further research is needed to validate and expand on these results, ultimately defining the optimal Pnp strategy for RARP and improving patient outcomes.

## Introduction

The effect of pneumoperitoneum (Pnp) on kidney physiology has become an area of investigation, particularly as laparoscopic and robot-assisted surgical procedures continue to gain prominence. Robot-assisted radical prostatectomy (RARP) has become the gold standard in surgery for localised prostate cancer. Compared to open radical prostatectomy, RARP has a higher risk of developing transient acute kidney injury (AKI). [1]

The incidence of AKI after RARP contributes primarily to the Pnp that is believed to be associated with renal changes. The mechanism behind this is not well understood. But it is likely to be caused by direct compression of Pnp on renal parenchyma and vasculature, leading to increased vascular resistance, venous and lymphatic congestion, and decreased renal blood flow. [2, 3] Renal autoregulation results in the stimulation of the renin–angiotensin–aldosterone system (RAAS), with increasing renin release and subsequent aldosterone secretion. Second, neuroendocrine responses result in the excretion of the anti-diuretic hormone. This results in salt and water retention with oliguria and a vicious cycle of renal cortical vasoconstriction leading to further activation of the RAAS [4].

These changes results in reduced renal blood flow and glomerular filtration rate, which can have implications for patient outcomes. As such, understanding the impact of pneumoperitoneum pressure on various biomarkers associated with kidney function is crucial in augmenting surgical techniques and minimising potential adverse effects on renal function.

In recent years, several kidney biomarkers have been identified as useful indicators of renal function and potential predictors of kidney injury. These biomarkers include Neutrophil Gelatinase-Associated Lipocalin (NGAL), Vascular Endothelial Growth Factor (VEGF), Osteoactivin, Kidney Injury Molecule-1 (KIM-1), Trefoil factor 3 (TFF3), Clusterin, and Calbindin. Each of these biomarkers plays a distinct role in kidney physiology and is expressed in the specific tubular or interstitial component of the kidney [5].

Urinary NGAL is a protein rapidly upregulated in response to kidney injury, serving as an early and sensitive marker for acute kidney injury (AKI). It has been shown to predict the severity of renal dysfunction [6–8].

Given the importance of these biomarkers in assessing renal function and injury, understanding how different pneumoperitoneum pressures impact their levels during RARP is vital. This study aimed to investigate the effect of low Pnp on renal function urinary biomarkers through a randomised clinical trial. The results are expected to offer valuable insights into the potential impact of pneumoperitoneum on renal function and inform surgical practice, thereby minimising adverse renal effects during laparoscopic procedures.

To our knowledge, this is the first study investigating the effect of pneumoperitoneum on urinary kidney injury markers.

## Methods

## Materials and methods

### Study design and participants

This study was a single-centre, triple-blinded, randomised clinical trial. We aimed to investigate the effect of low Pnp on renal function for patients with prostate cancer who underwent robot-assisted radical prostatectomy (RARP) at the Department of Urology, Aalborg University Hospital, Aalborg, Denmark. Eligible patients aged between 40 and 75 years old with previously untreated, histologically confirmed, focal prostate cancer who were offered RARP. Patients were ineligible if they were not able to give informed consent, complete trial documentation, or speak or understand the Danish language.

The study complied with the principles of the Declaration of Helsinki and was approved by the Ethics Committee of the North Denmark Region (N-20200078, 08. December 2020) and the Danish Data Protection Agency (2020-118, 28. September 2020), and registered at ClinicalTrials.gov (NCT04755452, 16. February 2021). All patients provided written informed consent, and the trial proceeded according to Good Clinical Practice and CONSORT guidelines [9]**.**

We used RedCap (Research Electronic Data Capture) software provided by Aalborg University Hospital to collect data and perform web-based randomisation.

### Procedure

To reduce surgical heterogeneity and ensure minimal dropout from the intervention group, the procedures were performed by two high-volume surgeons, each of whom had carried out at least 300 RARPs prior to the trial. All surgeries were conducted in the steep Trendelenburg position, and as described by Huynh et al.[10] After administering general anaesthesia, the operating department practitioner (ODP) inserted a urinary catheter before starting the surgery. They collected 20 mL of urine and documented intraoperative urine production. The dorsal venous complex (DVC) was handled using a suture ligation method, and to maintain consistency throughout the procedure, the pneumoperitoneum pressure (Pnp) was kept constant during DVC dissection.

Nurses in the ward recorded urine production postoperatively. On the next day, as the patient prepared for discharge, they collected another 20 mL of urine before the patient left the ward. The samples were stored at – 80 °C for future analysis of urinary kidney injury markers. Blood tests for serum creatinine were conducted one week before surgery, on the 1st postoperative day (POD), and lastly, at 10th POD.

### Randomisation and masking

RedCap (Research Electronic Data Capture) software, hosted by Aalborg University Hospital, was used to collect data and conduct web-based randomisation. The patients were randomly assigned in a 1:1 ratio into a low Pnp (7 mmHg) or a standard Pnp group (12 mmHg). The primary researcher organised the randomisation process and completed it while the patient was in the theatre. After the patient was anaesthetised, the sealed envelope containing the allocation group was handed over to the ODP in the operating room. The surgeon was advised to set all surgical ports at Pnp = 10–12 mmHg. After the surgeon completed port placement and robot docking, the ODP opened the envelope and adjusted the Pnp according to the assigned pressure group. The nurse covered the pressure indicator in the insufflator to ensure that the surgeon was unaware of the assigned pressure group and maintain the blinding measures. Medical laboratory technologists were also included in the blinding measures and remained uninformed of the patients’ group allocations and randomisation.

### Outcomes

The primary outcome is the change in urinary Neutrophil Gelatinase-Associated Lipocalin (u-NGAL) before and after surgery. Secondary outcomes include changes in urinary markers that represent all nephron segments, urinary electrolytes, creatinine, and albumin. We also assessed the risk of AKI according to the standard; Kidney Disease: Improving Global Outcomes (KDIGO) criteria [11].

### Analysis of urine samples

The Human Lipocalin-2/NGAL Quantikine ELISA Kit from RnD (Biotechne, UK, Cat # DLCN20, lot # P306758) was used to determine u-NGAL as described by the manufacturer.. Samples were analysed in singlets, diluted 5–20 fold. Controls were included at each run. Inter-assay coefficients of variation (CVs) in 7 runs were 6.1% (level 0.9 ng/mL), 3.5% (level 2.8 ng/mL), and 5.4% (level 5.5 ng/mL). Pre- and post-surgery samples were analysed on the same plate.

The kidney injury markers Calbindin, Clusterin, KIM-1, Osteoactivin, TFF3, and VEGF were analysed by the ELISA multiplex assay (Kidney Injury Panel 3 (human) Kit Cat# K15189D-1 Lot# K0040523, Meso Scale Discovery (MSD), Rockville, USA) according to the manufacturer’s protocol. In short, the 96-well precoated plates were incubated with blocking buffer and washed 3 times with premade wash buffer: PBS (Lonza Cat#17–512-F), 0.05% Tween-20 (Sigma P9416-100ml) at an ELx50 BioTek plate was before analysis. Urine samples were thawed, mixed, and subjected to a quick spin at 17000g and diluted 1:10 in 96-well dilution plates in singlets, together with standards and KIM-1 controls (R&D systems, cat# C24, Lot# P292291) in duplicate. Fifty µl of samples, standards, and controls were transferred to the MSD plate and incubated for 2 h, washed, and incubated with detection antibody for 2 h. Subsequently, the plate was washed, and read buffer was added. The plates were immediately read at a MESO QuickPlex SQ120MM Reader. Pre- and post-surgery samples were analysed on the same plate.

For analysis of the results, we used MSD Discovery Workbench version 4.0. Standard curves were generated from serially diluted calibrator by 4-parameter logistic regression. Nine samples obtained values either above or below the detection range for KIM-1, Clusterin, VEGF and/or Calbindin and were repeated in appropriate dilutions or undiluted. Out of 177 results, 103 were below the detection limit for TFF3 in dilution 1:10, and on this background, results for TFF3 are not reported. KIM-1 control results were used for the calculation of intra- and inter-assay CV. Mean intra-assay CV was 5.1% (level 4834.9 pg/ml, *N* = 3 duplicates) and 3.3% (level 1548.5 pg/ml, *N* = 3 duplicates), and inter-assay CV’s were 14.3% and 10.8%, respectively (*N* = 3 plates). The concentrations of potassium, sodium, chloride, creatinine, and albumin in urine were measured on a Cobas 6000 (Roche, Germany) at the Clinical Biochemistry Department..

### Statistics

All analyses were based on the intention-to-treat population. Mean, standard deviation (SD), and 95% confidence interval (CI) or median and interquartile range (IQR) were reported for continuous data as appropriate, while categorical data were presented as *n* (%).

A repeated measures model using a robust variance estimate was used to estimate mean differences and 95% confidence intervals for the urinary kidney injury biomarkers [12]. No imputation of missing data was performed.

Between-group differences were evaluated using the chi-squared or Fisher exact tests for categorical variables and the *t* test and nonparametric Mann–Whitney *U* test for continuous variables. All analyses were conducted using STATA (version 17).

## Results

**Fig. 1:**
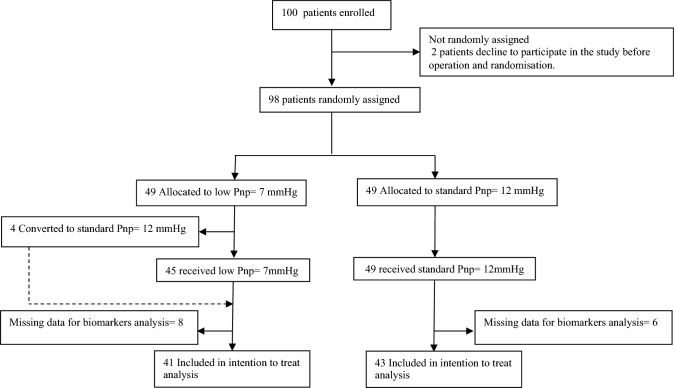
Trial profile

Between 2nd March 2021 and 28th January 2022, 98 patients were randomly assigned and allocated to either a standard Pnp of 12 mmHg (*n* = 49) or a low Pnp of 7 mmHg (*n* = 49) during robot-assisted radical prostatectomy (RARP). However, four patients (8%) from the low Pnp group necessitated an elevation of Pnp to 12 mmHg for longer than 20 min due to inadequate workspace or bleeding. Despite this adjustment, these patients were maintained in the low Pnp group as per the intention-to-treat principle for subsequent analysis, trial profile (Fig. 1).

Baseline patient characteristics and demographics are described in Table 1.

**Table 1:** Baseline characteristics of patients

	Standard Pnp (*n* = 49	Low Pnp (*n* = 49)
Age, year	66.9 (6.3)	65.5 (7.0)
BMI	27.1 (2.8)	28.0 (3.5)
Hypertension	19 (38.8%)	22 (44.9%)
DM	5 (10.2%)	4 (8.2%)
Previous abdominal surgery	5 (10.2%)	6 (12.2%)
PSA	11.1 (7.5)	10.3 (6.7)
Prostate volume, ml	54.3 (25.7)	51.8 (28.9)
T stage from DRE		
T1c	24 (49.0%)	28 (57.1%)
T2a	9 (18.4%)	11 (22.4%)
T2b	12 (24.5%)	6 (12.2%)
T2c	4 (8.2%)	4 (8.2%)
ISUP grade		
2	16 (32.7%)	19 (38.8%)
3	16 (32.7%)	12 (24.5%)
4	3 (6.1%)	4 (8.2%)
5	14 (28.6%)	14 (28.6%)

Data presented as mean (SD) for continuous variables, and as n (%) for categorical variables. There is no baseline missing data

*PSA* prostate specific antigen, *DRE* digital rectal examination, *ISUP* grade International Society of Urologic Pathologists

Postoperatively, patients in the low Pnp group demonstrated significantly lower levels of NGAL compared to the standard Pnp group (mean difference − 39.9, 95% CI − 73.7 to − 6.1, *p* = 0.02). No significant differences were observed for other urinary biomarkers (Table 2), nor for urinary electrolyte and Albumin Creatinine Ratio (ACR) (Table 3).

**Table 2:** Urinary biomarkers in robot-assisted radical prostatectomy (RARP) patients: comparing low = 7 mmHg vs standard = 12 mmHg pneumoperitoneum (Pnp)

		After surgery: mean	sd	Before surgery: mean	sd	Effect: Diff from 12 mmHg	[95% CI]		*p* value
NGAL (ng/ml)	12 mmHg	60.9	109.5	22.5	35.2				
	7 mmHg	22.8	21.5	22.5	35.2	− 39.9	− 73.7	− 6.1	0.02
VEGF (pg/ml)	12 mmHg	1109.9	1837.5	2393.5	5115.1				
	7 mmHg	617.5	536.6	2393.5	5115.1	− 492.4	− 1067.5	82.7	0.09
Osteoactivin (pg/ml)	12 mmHg	10,294.9	9770.4	3011.8	2515.5				
	7 mmHg	7160.0	5030.4	3011.8	2515.5	− 3105.4	− 6394.3	183.6	0.06
KIM-1 (pg/ml)	12 mmHg	1809.1	1303.2	474.6	437.2				
	7 mmHg	1950.0	2203.6	474.6	437.2	145.5	− 616.3	907.3	0.71
Clusterin (pg/ml)	12 mmHg	218,158.5	228,839.5	28,055.1	60,547.5				
	7 mmHg	224,658.7	389,323.9	28,055.1	60,547.5	6710.4	− 128,957.0	142,377.8	0.92
Calbindin (pg/ml)	12 mmHg	32,585.9	32,088.9	14,389.3	26,922.6				
	7 mmHg	26,270.9	35,330.9	14,389.3	26,922.6	− 4654.0	− 18,497.1	9189.2	0.51

This table presents the mean and standard deviation (SD) of various urinary biomarkers, including neutrophil gelatinase-associated lipocalin (NGAL), vascular endothelial growth factor (VEGF), osteoactivin, kidney injury molecule-1 (KIM-1), clusterin, and calbindin, measured before and after RARP in patients who underwent surgery with either 7 mmHg or 12 mmHg pneumoperitoneum. The table also provides the mean difference between the two groups, along with their 95% confidence intervals (CI), and p values to determine the statistical significance of the differences

**Table 3:** Urinary electrolyte analysis in robot-assisted radical prostatectomy (RARP) patients: comparing Low = 7 mmHg vs. Standard = 12 mmHg pneumoperitoneum

	Low Pnp (95% CI)	Standard Pnp (95% CI)	Difference from standard Pnp (95% CI)	*p*
U. Cloride	36.3 (31.1–41.4)	42.8 (34.7–50.8)	− 6.5 (− 15.9 to 2.9)	0.17
U. K	38.8 (31–46.6)	43.6 (36.2–51)	− 4.8 (− 15 to 5.5)	0.35
U. Na	32.5 (27.7–37.4)	39 (31.7–46.3)	− 6.4 (− 15 to 2.1)	0.13
ACR	1.9 (1.3–2.5)	2 (1.3–2.6)	− 0.03(− 0.9 to 0.8)	0.93

This table presents the mean and 95% confidence interval (CI) of various urinary electrolyte parameters, including urinary chloride (U. Chloride, mmol/L), urinary potassium (U. K, mmol/L), and urinary sodium (U. Na, mmol/L), as well as the albumin-to-creatinine ratio (ACR, mg/mmol) for patients undergoing robot-assisted radical prostatectomy (RARP)with either low or standard Pnp. The differences between the two groups, along with their 95% CI, and p values, are provided to determine the statistical significance of the differences

A significant difference was found in intraoperative urine production (median [IQR]: 100 [50–200] for standard Pnp vs 200 [100–325] for low Pnp, *p* = 0.01) and total postoperative urine production (median [IQR]: 1000 [850–1287] for standard Pnp vs 1325 [1025–1800] for low Pnp, *p* = 0.001). No significant differences were observed for other renal function parameters (Table 4).

**Table 4:** Renal function parameters in robot-assisted radical prostatectomy (RARP) patients: comparing Low = 7 mmHg vs. Standard = 12 mmHg pneumoperitoneum

	Standard Pnp	Low Pnp	*p*
Intra-op Urine Prod	100 (50–200)	200 (100–325)	0.01
I.V. fluid infusion	1418 (341)	1474 (412)	0.46
2-h Urine Prod. Post-surgery	200 (100–300)	300 (200–500)	0.0008
Total Urine Prod. (1POD)	1000 (850–1287)	1325 (1025–1800)	0.001
S.creatinine (1POD)	98.9 (25.2)	93.6 (25)	0.29
eGFR (1POD)	70.7(16.5)	74.8 (15.5)	0.20
S.creatinine (10POD)	88 (18.6)	82 (15.7)	0.08
eGFR (10POD)	76.4 (13.6)	80 (10.7)	0.14

This table presents the median and interquartile range (IQR) or mean and standard deviation (SD) of various intra-operative and post-operative parameters related to urine production, fluid infusion, and renal function in patients undergoing robot-assisted radical prostatectomy (RARP) with either standard or low Pnp. *p* values are calculated to determine the statistical significance of the differences between the two groups.

*IV* intra venous, *POD* post-operative day, *eGFR* estimated Glomerular Filtration Rate

The occurrence of AKI, defined according to the KDIGO criteria, was compared between patients. On the first day, AKI stage 1 was observed in 16 (32.6%) patients with low Pnp and 18 (36.7%) patients with standard Pnp, with no significant difference between the groups (*p* = 0.67). For AKI stage 2 on the 1st day, fewer patients developed this stage in the low Pnp group, with 2 (4%) patients compared to 5 (10%) patients in the standard Pnp group. However, this difference was not statistically significant (*p* = 0.43). By the 10th day post-operation, 6 (12%) patients in each group still had a symptomatic stage 1 AKI, with no significant difference between the groups (*p* = 1.0). According to the cutoff of u-NGAL reported in previous studies that can predict AKI stage 2/3 as 78ng/ml, [13] our results showed that 3 (7%) from low Pnp and 5 (11%) from standard Pnp developed AKI stage 2/3 (*p* = 0.73).

Urinary electrolytes and albumin-to-creatinine ratio (ACR) did not differ significantly between the study groups (Table 3).

## Discussion

To the best of our knowledge, this study is the first to investigate the effect of low Pnp on renal function utilising a spectrum of urinary biomarkers.

We observed a stable level of u-NGAL in the low Pnp group after surgery, which may be interpreted as minimal renal changes or milder effects within this group. In contrast, the standard Pnp group demonstrated an increase in u-NGAL levels, indicating a higher degree of renal impact. However, even this elevated level did not exceed the clinical threshold of 78 ng/ml, a previously identified cutoff value predictive of AKI [13]. Therefore, while the increase was statistically significant, it may not suggest a clinically meaningful difference.

The significant decrease in intra- and postoperative urine production in the standard Pnp group may have contributed to this mildly elevated u-NGAL.

In a somewhat contrasting observation, Filho et al.s study examining the effect of low Pnp during laparoscopic cholecystectomy, no significant difference in plasma NGAL was observed 24 h post-surgery between the standard (10–12 mmHg) and low Pnp (6–8 mmHg) groups [14]. This contrast with our findings could potentially be explained by the duration of surgery and the consequent exposure to Pnp. Filho et al. reported a duration of 70 min for the standard Pnp group and 77 min for the low Pnp group during laparoscopic cholecystectomy. In contrast, our more intricate procedure, radical prostatectomy, had a longer Pnp duration: 152 min for the standard Pnp group and 156 min for the low Pnp group.

These divergent findings are also corroborated by animal studies showing a strong correlation between increased NGAL levels and the duration of Pnp exposure. Notably, one study on rats documented significant increases in NGAL levels after the second hour of exposure to Pnp, a pattern consistent with our observations [15]. The study also showed that this increase was more pronounced at high Pnp. While serum creatinine levels remained unchanged, novel markers like NGAL presented a clear response to prolonged Pnp, underscoring their sensitivity in detecting renal injury.

The AKI detection using u-NGAL was well aligned with the KDIGO criteria in defining stage 2/3 AKI. Caution is necessary when interpreting these findings, as the lack of a notable difference in the gold standard definition of AKI by KDIGO between the two groups suggest that the observed variation in u-NGAL levels might not have a considerable influence on overall renal function or clinical outcomes. There are no changes in other kidney injury markers identified.

The renal epithelial biomarkers Calbindin, Clusterin, KIM-1, Osteoactivin (OA), and VEGF are known to indicate kidney damage and display elevated levels in urine across various kidney disorders [16, 17]. Calbindin is an extracellular Ca^2+^ binding protein that is primarily expressed by distal tubular and collecting duct cells [18, 19]. It is linked to distal tubular cell damage, and exhibits increased expression in vitro following exposure to agents like cisplatin [20]. Studies showed a strong correlation between urinary calbindin levels and AKI. [17, 21] OA, also known as glycoprotein non-melanoma clone B (gpnmb), is a protein that plays a crucial role in the differentiation and functioning of various cell types. The therapeutic potential of OA has been explored in tissue regeneration for bone defects, liver damage, muscle atrophy, and kidney injury. [22] High expression of OA in the tubular epithelial cells and renal interstitium was identified in the animal model after unilateral ureteral obstruction [23].

In cases of acute kidney injury (AKI) resulting from intrinsic renal causes, significant proximal tubular injury can occur. This tubular injury may hinder the proximal tubule's ability to reabsorb albumin, leading to albuminuria [24]. Prior research has demonstrated that the urinary Albumin–Creatinine Ratio (u-ACR) serves as a sensitive biomarker for detecting AKI, and it can predict the risk of AKI earlier than serum creatinine levels alone [25, 26].

Considering the current findings, the clinical implications of using low Pnp during RARP merit further exploration. The reduced u-NGAL levels observed in the low Pnp group may suggest a potential role for low Pnp in minimising renal injury and promoting faster recovery. The lack of a notable difference in the gold standard definition of AKI by KDIGO between the two groups suggests that the clinical impact may be limited. It is valuable to investigate if specific patient populations, such as those with pre-existing renal conditions or during more complex and lengthy procedures such as radical cystectomy, will benefit more from low Pnp.

Our study has several limitations. The single-centre design may limit the generalisability of the results, and future multicenter studies with larger sample sizes may provide more robust evidence. Additionally, the short follow-up period may not be sufficient to detect any long-term effects of low Pnp on renal function.

Future research needs to focus on addressing these limitations and further investigate the effect of low Pnp on renal function during RARP. Long-term follow-up studies may reveal more pronounced differences in renal function between the two Pnp groups.

In conclusion, this study demonstrated low Pnp during RARP resulted in lower u-NGAL levels, suggesting a potential benefit in terms of reduced renal injury. However, the lack of a difference in AKI as defined by the KDIGO criteria between the groups indicates that the clinical significance of this finding may be limited. Further research is needed to validate and expand on these results, ultimately informing the optimal Pnp strategy for RARP and improving patient outcomes.

## Data Availability

Data are available upon reasonable request.
